# Prognostic value of cerebrospinal fluid free fatty acid levels in patients with acute ischemic stroke

**DOI:** 10.3389/fnhum.2015.00402

**Published:** 2015-07-14

**Authors:** Xue-Jun Wei, Meng Han, Guang-Chen Wei, Chong-Hao Duan

**Affiliations:** ^1^Department of Intensive Care Unit, The People’s Hospital of Laiwu CityLaiwu, China; ^2^Department of Emergency, The People’s Hospital of Laiwu CityLaiwu, China

**Keywords:** free fatty acid, acute ischemic stroke, cerebrospinal fluid, prognostic, Chinese, short-term

## Abstract

In this study, prognostic value of cerebrospinal fluid (CSF) free fatty acid (FFA) levels in patients confirmed with acute ischemic stroke (AIS) was evaluated in a Chinese population. A prospective cohort designed study was conducted at our hospital of the Emergency department from November, 2012 to September, 2014. The National Institutes of Health Stroke Scale (NIHSS) score on admission was applied to assess CSF levels of FFA and specific severity degree of stroke. Evaluation of the prognostic outcomes of those stroke patients used the modified Rankin scale scores at 90-days. Logistic regression analysis analyzed the prognostic value of FFA. NIHSS score results suggested a positive relationship between levels of CSF FFA levels and severity of stroke. There was an obviously higher trend of CSF FFA levels in patients with CE stroke than those of the non-CE stroke patients, with statistically difference (*P* < 0.05). Further, CSF FFA levels were evidently lower in those 73 patients with favorable outcome when compared to those with unfavorable outcomes [0.21(IQR, 0.11–0.28) mmol/L vs. 0.36 (IQR, 0.27–0.50) mmol/L, *P* < 0.0001, *P* < 0.0001]. Multivariate analysis results after possible confounders adjustment indicated that there was an increased risk of unfavorable outcome associated with CSF FFA levels ≥0.29 mmol/L (OR 5.12, 95%CI: 2.35–10.28; *P* < 0.0001). Collectively, CSF level of FFA at admission was suggested to be a useful, independent short-term prognostic marker in Chinese patient with AIS.

## Introduction

Stroke is a leading cause of mortality and subsequent serious long-term disability among survivors (Zhang and Zhang, 2014). During the recent decades, optimization of medical care and allocation of health care resources play critical roles in maintaining and increasing the survival outcomes of those patients, of which rapidly measurable biomarkers identification are extremely essential (Tu et al., 2013). Novel biomarkers have emerged to assist clinicians with decision-making.

In the post absorptive state, free fatty acids (FFAs) are the preferred fuel source for the heart, liver, and skeletal muscle, and yielding large quantities of adenosine triphosphate (ATP; Ebbert and Jensen, 2013). FFA enters the circulatory system from lipolysis of triglycerides stored in adipocytes, and the plasma FFA concentration is closely connected with lipid metabolism (Choi et al., 2014). Elevated FFA concentration is linked to several risk factors for atherosclerosis, including ischemic stroke risk (Yaemsiri et al., 2013). Interestingly, Seo et al. (2011) reported that elevated concentration of FFA might be one of the risk factors that contribute to the development of cardioembolic (CE) stroke.

Pathological processes, including ischemia, and subarachnoid hemorrhage could apparently lead to the increased cerebrospinal fluid (CSF) concentrations of FFAs, as supported by previous animal designed experimental evidence (Gasparovic et al., 2001). FFA accumulation begins within a few minutes of the onset of insult and has been shown to remain elevated for hours to days (Pilitsis et al., 2003). FFA accumulation can result in an uncoupling of oxidative phosphorylation and the formation of inflammatory by-products including reactive oxygen species (Nakamura et al., 2009). Despite the well-documented increases in FFAs in animal studies, FFA concentrations following stroke in humans have not been well studied. To our knowledge, there is still none relevant studies evaluated CSF FFA as a prognostic marker after stroke in our Chinese population. Hence, we conducted this prospective designed study for the purpose of evaluating the prognostic value of CSF FFA levels in patients confirmed with acute ischemic stroke (AIS) in the Chinese populations. In addition, whether FFA levels add significant clue to global prognostic indicator like NIHSS was also tested.

## Materials and Methods

Prospectively, this study was performed within the department of Emergency of Laiwu City People’s Hospital. From November, 2012 to September, 2014, all eligible patients with first-ever AIS were included in the present experiment. All patients were examined by MRI with diffusion-weighted imaging (DWI). And available data were carefully and completely recorded for further investigation. Baseline characteristics, including ages, genders, and history of conventional vascular risk factors such as hypertension, diabetes mellitus, hypercholesterolemia, coronary heart disease, atrial fibrillation (AF) were obtained; previous cigarette smoking habit was also recorded. Clinically, the use of stroke scale-the National Institutes of Health Stroke Scale (NIHSS) was applied for the assessment of stroke severity executed by a neurologist in hospital (Brott et al., 1989). For a reliable improvement of the classification of stroke subgroups, the Trial of Org 10172 in Acute Stroke Treatment (TOAST) criteria was used (Adams et al., 1993). By applying the Oxfordshire Community Stroke Project criteria, clinical syndrome of stroke was determined (Bamford et al., 1991).

Additionally, exclusion criteria of this study were predefined as follows: (1) patients who were previously diagnosed with malignancy, acute or chronic inflammatory diseases; (2) the patients who presented to the hospital beyond 24 h following stroke onset; (3) patients who without a brain imaging (MRI) test. All enrolled subjects were gave written informed consent, or by their family, and legal guardian. Institutional Review Board of Laiwu City People’s Hospital was contacted to accept the performance of this experiment.

The measurements of included subjects infarct volume was according to the formula 0.5 × a × b × c (a, the maximal longitudinal diameter, b, the maximal transverse diameter perpendicular to a, and c, the number of 10-mm slices containing infarct; Sims et al., 2009). Modified Rankin Scale (mRS) score was used for the judgment of functional outcomes, which was predefined to be obtained on days 90. Detailed scores of this criteria range from 0 to 6 blinded to FFA levels, where a higher score indicate increasing severity (Bonita, 1988). Month 3 (90 days) was the primary end point of the study, focusing on the exploration of the favorable functional outcome of stroke patients. And the secondary end point was death due to any causes within a 90-day follow-up. Two trained medical students were selected to execute the assessment of outcomes of this trial, through a structured telephone interview. And concordance on most variables should be obtained by the two students. Reliability analysis of the mRS (internal consistency) yielded Cronbach’s α of 0.89 in this study.

Cerebrospinal fluid samples of inclusions were obtained at admission. CSF aliquots were centrifuged at 4000 rpm for 10 min and then frozen at -70°C until assayed. The CSF FFA was measured using an enzyme cycling method by OLYMPUS AU2700 (OLYMPUS, Tokyo, Japan; intra-assay coefficient of variation [CV] 1.03–1.22%, inter-assay CV 1.32–1.45%). The detection upper and lower limits were 3.00 and 0.01 mmol/L, respectively. In order to test the relationship between FFA and inflammation, CSF levels of high sensitivity C-reactive protein (Hs-CRP) were also tested (Stearman and Southgate, 1994; Takizawa et al., 2001; Is et al., 2007). And just in case, standard for these undetectable levels was preset to equal to the lower detection limit.

In this study, discrete variables are expressed as percentage or frequencies and continuous variables as medians with interquartile range (IQR). The application of the χ^2^ test (Chi-square test) was used for the comparison of proportions, whereas the comparison of continuous variables applied the Mann–Whitney test. Spearman’s rank correlation was calculated to assess the relationship between CSF FFA and others factors. Further, multivariate logistic regression models was conducted to assess the associations between FFA and NIHSS score, infarct volume, as well as the influence of FFA levels on functional outcome, after the adjustment with possible confounders such as age, gender, time from onset to admission, stroke syndrome, stroke etiology, vascular risk factors, and CSF levels of Hs-CRP. The association between CSF FFA levels and AIS prognosis were expressed as adjusted odds ratios (OR) and 95% confidence interval (95%CI). The receiver operating characteristic (ROC) analysis was used to determine the optimal cut-off values from different predictive models for the comparison of different prognostic risk scores, combined with the calculation of area under the curve (AUC), which was selected as a summary measure over criteria and cut-point choices. Data were analyzed with SPSS software version 19.0 (SPSS Inc., Chicago, IL, USA) and the ROCR package (version 1.0-2; http://cran.r-project.org/). The difference was statistically significant when *P* < 0.05.

## Results

In our study, CSF was collected after admission for standardized measurement of FFA and Hs-CRP in 238 patients. Of the 238 patients, 145 (60.9%) were male with a median age of 64 years (IQR, 53–76). The median NIHSS score of patients was eight points (IQR, 5–13) on the admission. A total of 59 (24.8%) patients were confirmed as AF and 47 (19.7%) with a family history of stroke diseases. For the purpose of this study, CSF samples were drawn at admission [within 0–6 (*n* = 89), 6–12 (*n* = 95), and 12–24 (*n* = 54) hours from symptom onset]. The primary baseline characteristics were illustrated in **Table 1**.

**Table 1:** Baseline characteristics of acute ischemic stroke (AIS) patients and normal cases.

Characteristics	*N* = 238
Male sex (%)	145(60.9)
Age (years), median (IQR)	64(53–76)
Stroke severity, median NIHSS score (IQR)	8(5–13)
Infarct volume (mL, IQR)	25(8–44)
Vascular risk factors, %	
Diabetes mellitus	20.2
Hypertension	67.2
Hypercholesterolemia	30.3
Coronary heart disease	31.1
Atrial fibrillation	24.8
Family history for stroke	19.7
Smoking habit	21.0
Laboratory CSF findings (median, IQR)	
FFA (at admission, mmol L^-1^)	0.24(0.15–0.34)
Hs-CRP (at admission, mg dL^-1^)	0.12(0.07–0.19)
Stroke etiology, %	
Small-vessel occlusive	21.0
Large-vessel occlusive	16.8
Cardioembolic (CE)	35.3
Other	14.3
Unknown	12.6
Stroke syndrome, %	
TACS	34.5
PACS	23.5
LACS	20.2
POCS	21.8

*IQR, interquartile range; TACS, total anterior circulation syndrome; LACS, lacunar syndrome; PACS, partial anterior circulation syndrome; POCS, posterior circulation syndrome; NIHSS, National Institutes of Health Stroke Scale; Hs-CRP, high C-reactive protein.*

**FIGURE 1:**
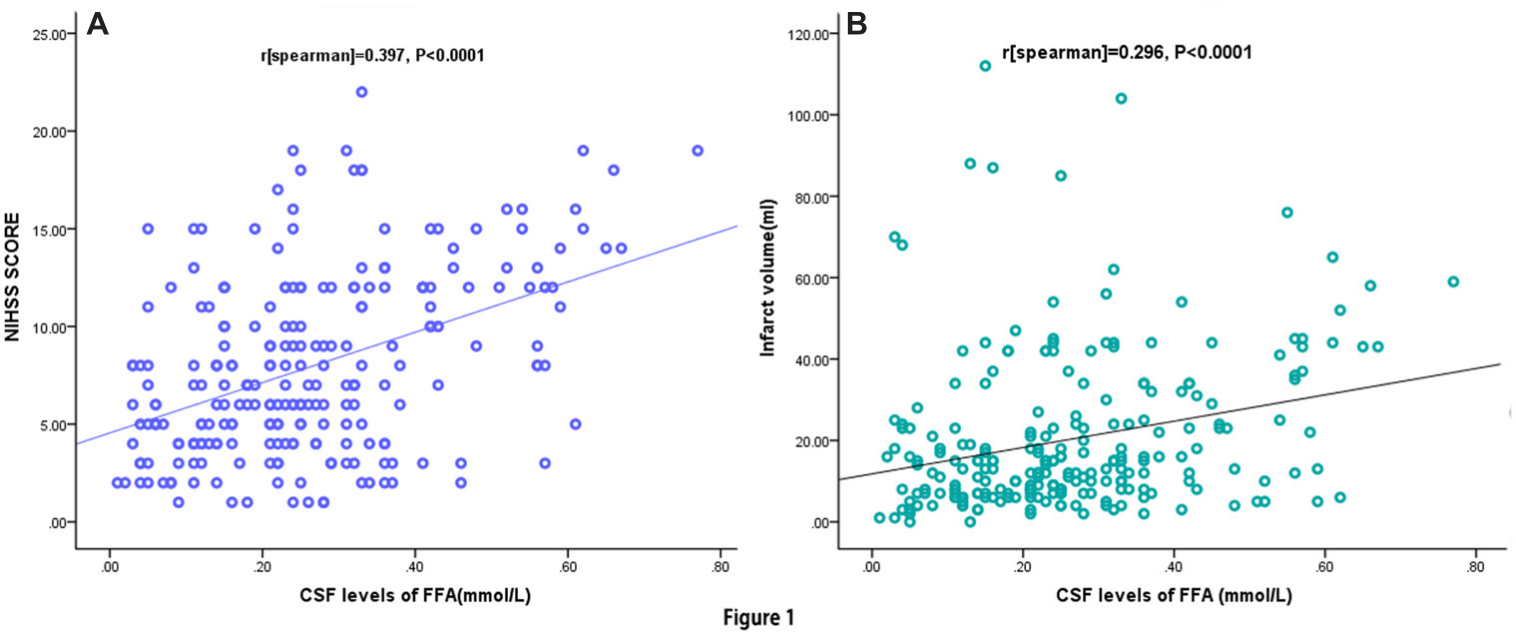
**Correlation between CSF FFA levels and others predictors. (A)** Correlation between CSF FFA levels and the National Institutes of Health Stroke Scale (NIHSS) score; **(B)** Correlation between CSF FFA levels and infract volume. CSF, cerebrospinal fluid; FFA, free fatty acid.

The increase in CSF FFA levels correlated with increasing disease severity of stroke based on the NIHSS score. The levels of FFA in CSF were positively linked with the NIHSS score (*r* = 0.397, *P* < 0.0001; **Figure 1A**) and Hs-CRP levels (*r* = 0.221, *P* = 0.002). Multiple logistic regression analysis results showed that a significant positive trend was observed between the FFA levels and the NIHSS score (*P* = 0.003). Additionally, CSF FFA levels were also positively related to the infarct volume (*r* = 0.296, *P* < 0.0001; **Figure 1B**). Meanwhile, the multiple logistic regression analysis results indicated that a significant positive trend was persisted between the FFA levels and the infarct volume (*P* = 0.009). Interestingly, we also found that FFA levels were positively connected with the time from stroke onset to admission (*r* = 0.186, *P* = 0.024). The correlation between stroke category and FFA levels were assessed. CSF FFA levels in patients with CE stroke were significantly higher as compared with non-CE stroke [0.37mmol/L (IQR, 0.27–0.45) vs. 0.15 mmol/L (IQR, 0.07–0.21); *P* < 0.0001]. Statistical analysis revealed that there was no significant influence of gender, age, stroke syndrome, and other risk factors of stroke on FFA in AIS patients (*P* > 0.05, respectively). Interesting, we found that the patients with AF had elevated CSF FFA levels than others [0.33 (IQR, 0.24–0.45) mmol/L vs. 0.24 (IQR, 0.13–0.33) mmol/L, *P* < 0.0001].

**FIGURE 2:**
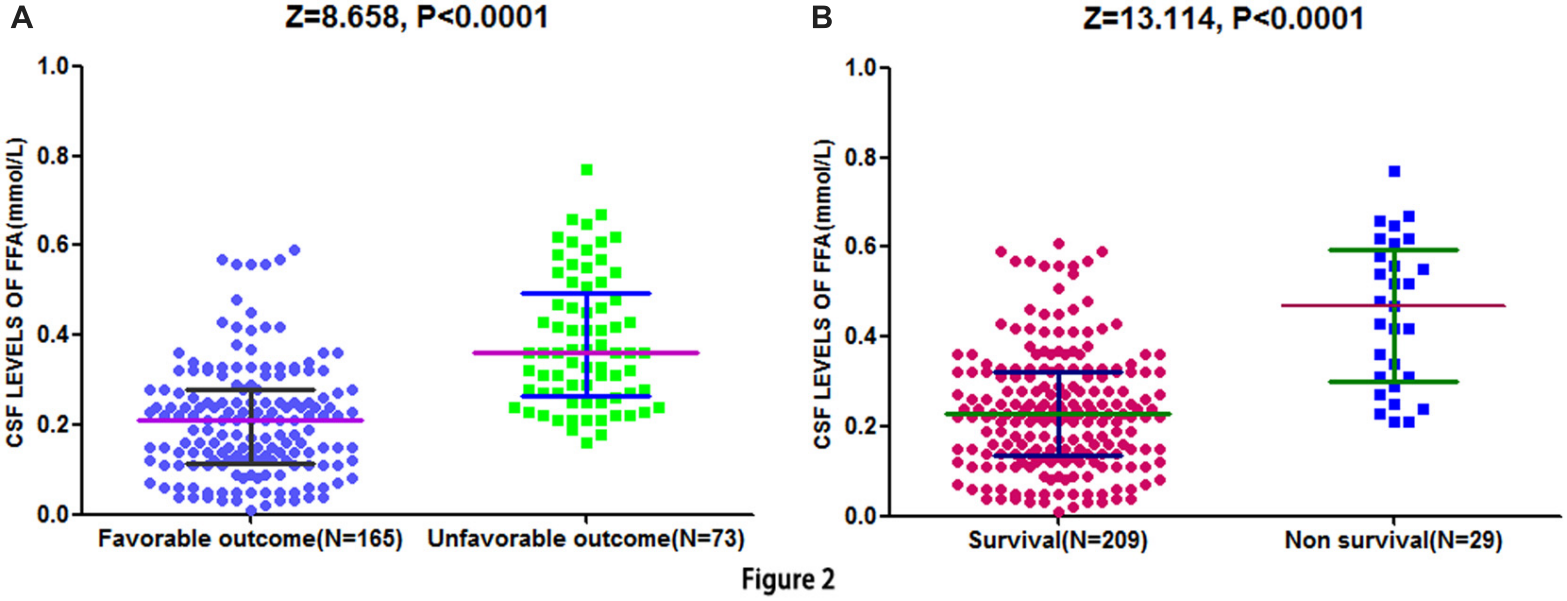
**Distribution of CSF FFA levels in different groups. (A)** Distribution of CSF FFA levels in stroke patients with favorable and unfavorable outcome. **(B)** Distribution of CSF FFA levels in patients with survival and non-survival. The horizontal lines indicate medians and interquartile ranges (IQRs) levels. *P*-values refer to Mann–Whitney *U* tests for differences between groups. CSF, cerebrospinal fluid; FFA, free fatty acid.

At 90-day follow-up, 73 out of 238 patients (30.7%) showed an unsatisfactory functional outcome and the median mRS score was 4 (IQR, 3–6). Compared to patients with favorable outcomes, the patients with unfavorable outcomes had elevated CSF FFA levels [0.36 (IQR, 0.27–0.50) mmol/L vs. 0.21 (IQR, 0.11–0.28) mmol/L, *P* < 0.0001; **Figure 2A**]. ROC curve showed that the optimal cut-off value of CSF FFA levels was projected to be 0.29 mmol/L as an predictor for functional outcome, which achieved a sensitivity of 84.5% and specificity of 80.8%, and the area below the ROC curve was 0.825 (95%CI, 0.773–0.883). Based on the AUC of 0.825, FFA showed a markedly greater discriminatory ability when compared with the Hs-CRP (AUC, 0.668; 95% CI, 0.601–0.738; *P* < 0.001), the NIHSS score (AUC, 0.744; 95% CI, 0.667–0.811; *P* < 0.01), and infarct volume (AUC, 0.584; 95% CI, 0.503–0.665; *P* < 0.0001). Further, FFA may improve the patients’ NIHSS score (AUC of combined model, 0.858; 95% CI, 0.805–0.910; *P* < 0.001).

The study results also showed that CSF FFA levels ≥0.29 mmol/L was closely connected with an elevated risk of unfavorable outcomes for AIS patients (unjusted OR 7.27, 95% CI: 3.94–13.41; *P* < 0.0001). Multivariate analysis indicated that elevated risk of unfavorable outcomes was related to CSF FFA levels ≥0.29 mmol/L (OR 5.12, 95%CI: 2.35–10.28; *P* < 0.0001; **Table 2**). In addition, four other indicators, including NIHSS score, age, infarct volume, and Hs-CRP, remained to be significant predictors for functional outcome.

**Table 2:** Univariate and multivariate logistic regression analysis for outcome.

Predictor	Univariate analysis			Multivariate analysis		
	OR	95% CI	*P*	OR	95% CI	*P*
FFA (≥0.29 mmol/L)	7.27	3.94–13.41	<0.0001	5.12	2.35–10.28	<0.0001
Age (increase per unit)	1.21	1.05–1.88	0.004	1.06	1.01–1.22	0.001
Hs-CRP (≥ 0.16 mg/dL)	2.22	1.45–3.13	<0.0001	1.76	1.33–2.43	<0.0001
Infarct volume (increase per unit)	1.18	1.10–1.32	0.003	1.15	1.06–1.29	0.009
NIHSS (increase per unit)	1.55	1.30–1.86	<0.001	1.31	1.10–1.48	<0.001
Hypertension	1.92	1.14–3.19	0.011	1.55	1.02–2.98	0.144
Atrial fibrillation	1.62	1.00–2.70	0.064	–		
Hypercholesterolemia	0.78	0.48–1.27	0.323	–		
Coronary heart disease	1.20	0.75–1.95	0.464	–		
Small-vessel occlusive	0.61	0.21–1.80	0.376	–		
Large-vessel occlusive	1.05	0.68–1.62	0.845	–		
CE	1.12	0.74–1.69	0.742	–		
TACS	1.81	1.41–3.54	0.532	–		
PACS	1.78	1.21–3.99	0.743	–		
LACS	0.35	0.12–1.02	0.054	–		
POCS	0.66	0.29–1.48	0.302	–		

*OR, odds ratio; CI, confidence interval; Hs-CRP, high-sensitivity C-reactive protein; NIHSS, National Institutes of Health Stroke Scale; LACS, lacunar syndrome; PACS, partial anterior circulation syndrome; POCS, posterior circulation syndrome; TACS, total anterior circulation syndrome.*

Cerebrospinal fluid free fatty acid levels in 29 patients who died showed greater than twofold increase as compared to patients who survived [0.47 (IQR, 0.30–0.59) mmol/L vs. 0.23 (IQR, 0.14–0.31) mmol/L, *P* < 0.0001; **Figure 2B**]. Similarly, FFA level can still be acted as an independent indicator for predicting the mortality (OR 6.13, 95% CI, 3.30–12.07), after multivariate adjustment for the outcome predictors including age, NIHSS score, and Hs-CRP.

## Discussion

Previous animal study has demonstrated that the up-regulated levels of certain CSF biomarkers may reflect the pathological activities in central nervous system (CNS), and may be in relation to ischemic diseases (Hjalmarsson et al., 2014). However, only a few human clinical studies have assessed the associations of the CSF biomarkers with stroke. Several studies have assessed several peripheral indicators of brain damage, including amyloid precursor protein metabolites (Selnes et al., 2010), YKL-40 (Kaynar et al., 2005), myelin basic protein (Lamers et al., 2003), Neuron-specific enolase (Selakovic et al., 2005), protein S100B (Brouns et al., 2010), glial fibrillary acidic protein (Steiner et al., 2006), and Monocyte chemoattractant protein-1 (Losy and Zaremba, 2001). To our knowledge, we firstly evaluated the accuracy and predictive value of CSF FFA levels to the functional outcomes of AIS patients at 90-day after admission in Chinese sample. Our primary finding was that CSF FFA level was a meaningful and independent predicting indictor for functional outcome 90-day after ischemic stroke, and CSF FFA levels ≥0.29 mmol/L were linked to a 5.12-fold increase in unfavorable outcome. Importantly, FFA could improve prognostic value of NIHSS score for clinical outcome and was a better prognostic marker compared with other markers. Additionally, our study results also indicated that elevated CSF FFA levels was implicated in the increasing severity of stroke after accounting for the NIHSS score, and also the CSF FFA levels were both positively connected with the Hs-CRP levels in CSF and the infarct volume.

Seo et al. (2011) reported that the baseline plasma level of FFA in CE stroke patients was approximately 1.5-fold higher than that of non-CE stroke patients. In line with the previous study, we also found that CSF FFA levels in CE stroke patients were significantly higher as compared to non-CE stroke (*P* < 0.0001). In another study, Pilitsis et al. (2003) found that up-regulation CSF levels of polyunsaturated fatty acids (PUFAs) that obtained within 2 days of insult were connected with a worse clinical outcome at hospital discharge, after adjusting for the Glasgow Outcome Scale (*P* < 0.01). However, the small sample (*N* = 25) should be referred. In our study, we confirmed above conclusion in a larger sample.

Whether the high CSF FFA plays an independent role in contributing the prognosis or just an epiphenomenon to disease severity is uncertain. As we all know, a severe stroke is essentially implicated with a poor outcome. Nevertheless, several other reasons have been involved in the unfavorable outcome of AIS patients with higher FFA levels. First, FFA may generate arrhythmia. Numerous experimental studies have also suggested the arrhythmogenicity of FFA (Oliver and Opie, 1994; Khawaja et al., 2012). Previously, Seo et al. (2011) manifested that the connection between FFA and CE stroke might result from the association between FFA and AF. In ours study, we also found that patients with AF had elevated CSF FFA levels. Second, elevated FFA might have thrombogenicity in patients with CE stroke (Seo et al., 2011). Consistent with this, animal studies have demonstrated systemic thromboembolism after FFA infusion (Connor et al., 1963), with activation of factor XII by stearic acids postulated as a possible mechanism (Didisheim and Mibashan, 1963). Third, an increase of plasma FFA in stroke patients may reflect a metabolic state of CE stroke attributed to AF. Finally, the effects of FFA on brain inflammation have been evaluated (Gupta et al., 2012). Similarly, a positive correlation between FFA and Hs-CRP levels (*P* = 0.002) was found in our study. Dasu and Jialal (2011) concluded that FFA may aggravate the expression of HG-induced Toll-like receptors (TLRs) and can induce the production of monocytic cells with over superoxide release, which may activate the NF-κB activity and also induced pro-inflammatory factor release.

Several limitations of the current observational study should be noted while interpreting our results. Firstly, in our study, we did not measured the circulating CSF FFA levels, so it yielded no exactly data about when and how long the FFA levels were elevated in AIS patients. Nakano et al. (1990) observed an increase in FFA concentrations in the gerbil brain following 5 min of ischemia. This was followed by a slow increase in FFA levels that reached a peak at about 3 days and then declined once again. Secondly, the measurements of FFA levels were conducted after the stroke, which may not precisely reflect pre-stroke exposure. Thirdly, the study population was only composed of a single ethnic population. To better generalize our data, a long-term prospective cohort study with a larger population may be necessary. Lastly, the predictive values of circulating FFA levels on long-term functional outcome of AIS patients were not evaluated in this study protocol.

## Summary

CSF level of FFA at admission was a useful and independent short-term prognostic indicator in Chinese patient with AIS. Further studies should be conducted to investigate the underlying mechanism between CSF FFA levels and the poor outcome of AIS. In this regard, the prognosis of stroke patients in China may be improved after elucidate the underlying mechanisms, if possible.

## Conflict of Interest Statement

The authors declare that the research was conducted in the absence of any commercial or financial relationships that could be construed as a potential conflict of interest.
